# 4-*tert*-Butyl-2-[2-(1,3,3-trimethyl­indolin-2-yl­idene)ethyl­idene]cyclo­hexa­none

**DOI:** 10.1107/S1600536811014590

**Published:** 2011-04-29

**Authors:** Graeme J. Gainsford, Mohamed Ashraf, Andrew J. Kay

**Affiliations:** a Industrial Research Limited, PO Box 31-310, Lower Hutt, New Zealand

## Abstract

The title mol­ecule, C_23_H_31_NO, has two alternative cyclo­hexa­none configurations at the 4-position in a ratio of 0.663 (3):0.337 (3). The plane of the five-membered planar ring in the indolin-2-yl­idene subtends an angle of 2.19 (7)° with its fused aromatic ring, an angle of 16.24 (8)° with the plane of the major cyclo­hexa­none configuration and an angle of 8.54 (15)° with the bridging planar ethyl­idene C atoms. These last atoms subtend an angle of 8.37 (16)° with the mean plane through the major cyclo­hexa­none configuration. The mol­ecules pack approximately parallel to the (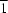
01) plane *via* C—H⋯π and C—H⋯O inter­actions.

## Related literature

For background information on potential applications of NLO (organic nonlinear optical material) compounds, see: Denk *et al.* (1990 ▶); Ma *et al.* (2002 ▶); Parthenopoulos & Rentzepis (1989 ▶). For synthesis details, see: Ainsworth (1963 ▶). For related compounds, see: Kawamata *et al.* (1998 ▶); Higham *et al.* (2010 ▶); Bhuiyan *et al.* (2011 ▶); Teshome *et al.* (2011 ▶). For the Cambridge Structural Database, see: Allen (2002 ▶). For graph-set notation of hydrogen bonds, see: Bernstein *et al.* (1995 ▶).
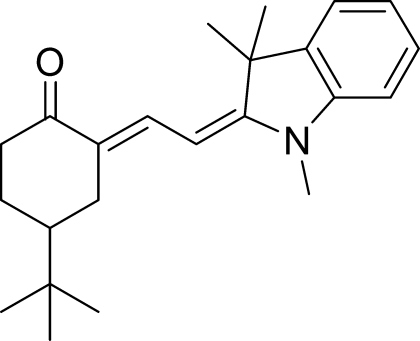


## Experimental

### 

#### Crystal data

C_23_H_31_NO
*M* *_r_* = 337.49
Monoclinic, 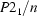

*a* = 9.7327 (4) Å
*b* = 17.2187 (6) Å
*c* = 12.1303 (4) Å
β = 100.045 (2)°
*V* = 2001.69 (13) Å^3^
*Z* = 4
Mo *K*α radiation
μ = 0.07 mm^−1^
*T* = 116 K
0.62 × 0.49 × 0.25 mm

#### Data collection

Bruker APEXII CCD diffractometer
Absorption correction: multi-scan (Blessing, 1995 ▶) *T*_min_ = 0.668, *T*_max_ = 0.746
44399 measured reflections
4500 independent reflections
3690 reflections with *I* > 2σ(*I*)
*R*_int_ = 0.043

#### Refinement

*R*[*F* ^2^ > 2σ(*F* ^2^)] = 0.050
*wR*(*F* ^2^) = 0.134
*S* = 1.05
4500 reflections
308 parameters
5 restraints
H atoms treated by a mixture of independent and constrained refinement
Δρ_max_ = 0.26 e Å^−3^
Δρ_min_ = −0.21 e Å^−3^

### 

Data collection: *APEX2* (Bruker, 2005 ▶); cell refinement: *SAINT* (Bruker, 2005 ▶); data reduction: *SAINT* and *SADABS* (Sheldrick, 1995 ▶); program(s) used to solve structure: *SHELXS97* (Sheldrick, 2008 ▶); program(s) used to refine structure: *SHELXL97* (Sheldrick, 2008 ▶); molecular graphics: *ORTEP-3* (Farrugia, 1997 ▶) and *Mercury* (Macrae *et al.*, 2008 ▶); software used to prepare material for publication: *SHELXL97* and *PLATON* (Spek, 2009 ▶).

## Supplementary Material

Crystal structure: contains datablocks global, I. DOI: 10.1107/S1600536811014590/jj2085sup1.cif

Structure factors: contains datablocks I. DOI: 10.1107/S1600536811014590/jj2085Isup2.hkl

Additional supplementary materials:  crystallographic information; 3D view; checkCIF report

## Figures and Tables

**Table 1:** Hydrogen-bond geometry (Å, °) *Cg*1 is the centroid of the C1–C6 ring.

*D*—H⋯*A*	*D*—H	H⋯*A*	*D*⋯*A*	*D*—H⋯*A*
C5—H5⋯O1^i^	0.95	2.45	3.3293 (19)	154
C9—H9*A*⋯*Cg*1^ii^	0.98	2.65	3.5705 (17)	156

Symmetry codes: (i) 
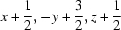
; (ii) 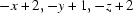.

**Fig. 1.**
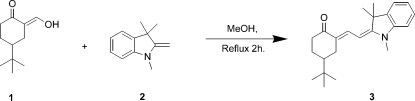
Reaction scheme showing the synthetic procedure for obtaining the title compound.

**Fig. 2.**
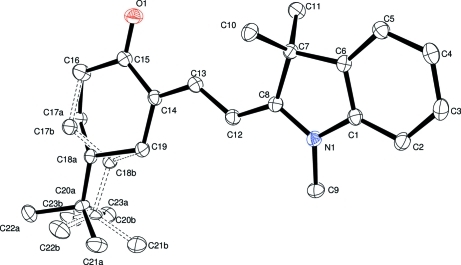
Molecular structure of the asymmetric unit (Farrugia, 1997); displacement ellipsoids are shown at the 30% probability level. H atoms not shown for clarity. Dashed bonds indicate positions of the minor (b) configuration atoms (see text).

**Fig. 3.**
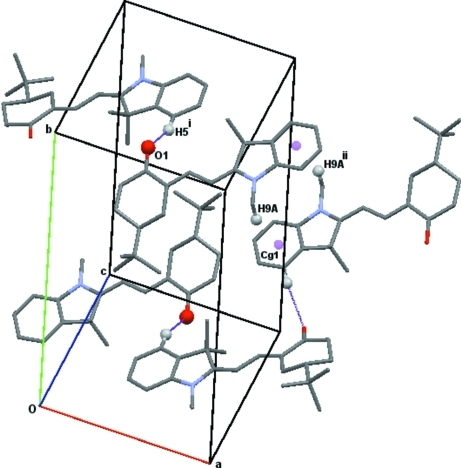
Partial packing diagram of the unit cell showing key interactions (see text and Table 1) [Macrae et al., 2008]. Only significant H atoms are shown as balls for clarity. Symmetry (i) x - 1/2, 3/2 - y, z - 1/2 (ii) 2 - x, 1 - y, 2 - z Two ring centres are shown as purple balls: Cg1 is the centre of the C1–C6 ring at symmetry (ii).

## supplementary crystallographic information

### Comment

Organic nonlinear optical (NLO) materials show much promise due to their potential application in areas such as optical power limiting, optical data storage and two-photon fluorescence imaging (Ma *et al.*, 2002; Parthenopoulos & Rentzepis, 1989; Denk *et al.*, 1990). Such compounds are typically push–pull conjugated systems that can be modified by altering either the donor, acceptor or conjugated interconnect moieties. However these modifications can involve trade-offs insofar as improvements to the nonlinear optical properties typically result in compounds that are more complex to prepare, have lower stabilities and higher optical losses. Conjugated ketones are useful intermediates for increasing the chain length and/or substituting different donors and acceptors onto a basic chromophore backbone. This is because conjugated ketones are quite reactive species and are able to undergo a range of carbon–carbon double bond forming reactions including the Wittig reaction, Knoevenagel condensation and Peterson olefination. With this in mind, and in line with our ongoing work on the development of novel organic NLO compounds, we sought to prepare the title compound 3 using the method outlined in Fig. 1. This compound is a useful synthon for the preparation of a range of chromophore systems as it contains an electron-rich indoline donor unit and a conjugated ketone onto which a range of acceptors can be coupled. The title molecule 3 is conveniently prepared in excellent yield by the condensation of 4-*tert*-butyl-2-hydroxymethylenecyclohexanone 1 with Fisher's base 2. Compound 1 was prepared from 4-*tert*-butylcyclohexanone using the general procedure reported by Ainsworth (1963).

Compound REFCODES below are from the CSD (Version 5.32, with Feb. 2011 updates; Allen, 2002). In the title compound 3 (Fig. 2), the cyclohexanone ring exists in two configurations, *S* (C18*a*) and *R* (C18*b*), in the ratio a:b of 0.663 (3):0.337 (3). This model made chemical sense, was stable in refinement, with insignificant difference Fourier residual density. The data supported refinement in the centrosymmetric space group *P*2_1_/*n* even though there were 57 weak reflections (with intensities between values between 0.08 (2) and 0.87 (7)) that violated the n glide absence condition. Refinement in *P*2_1_ did not improve the fit significantly as would be expected with such weak contributing data, and gave some very large correlations between thermal and positional parameters of the n glide related molecules.

The closest comparable structures for the cyclochexanone section of the molecule is QADZUQ, 4-*tert*-butyl-2,6-bis(4-methylbenzylidene)cyclohexanone (Kawamata *et al.*, 1998) which has a mirror plane passing through the carbonyl, *tert*-butyl and their bound ring C atoms. A comparision of the cyclohexanone dimensions indicates that some electronic delocalization along the ethylidene chain is observed with the C14—C15 bond length shortened (1.472 (2), 1.490 Å), the C13—C14 bond length lengthened (1.3619 (18), 1.332 Å) and the C12—C13 bond shortened (1.4204 (19), 1.466 Å) for 3 and QADZUQ respectively. The dienone-ether macrocyclic compound WUYMIN (Higham *et al.*, 2010) also contains copies of the 4-*tert*-butyl-cyclohexanone at a lower resolution (*R* 9.0%), with similar configurational disorder in the ratio of 0.70:0.30 as observed here.

As noted before in related indoline-based compounds there is minor buckling between the planar 5- and 6-membered rings in the indolin-2-ylidene ring of 2.19 (7)° compared with 1.81 (13)° in compound 17 (Teshome *et al.*, 2011) and 1.38 (9)° in compound TMIPI (Bhuiyan *et al.*, 2011). The interplanar angles confirm the consistent twist along the electronic delocalization plane: 8.54 (15)° between the 5-membered indoline ring (N1,C1,C6–C8) and the ethylidene atoms plane (C8,C12–C14) with a further 8.37 (16)° angle subtended between the latter and the average plane through the major configuration cyclohexanone atoms (C14–C16, C17*a*, C18*a* and C19). The indoline dimensions are identical to those found in the above-listed compounds.

The molecules are held in the lattice by weak C—H···π interactions (Table 1) over cell inversion centres and C—H···O hydrogen bonds, the latter forming C(10) motifs (Bernstein *et al.*, 1995), Fig. 3.

### Experimental

To a stirred solution of Fisher's base 2 (0.865 g, 5 mmole) in methanol was added compound 1 (0.91 g, 5 mmole). The mixture was refluxed for 2 h by which time its colour had changed from deep red to brown. The solvent was removed at reduced pressure and the residue purified by crystallization in ethanol, giving the title compound 3 as a yellow solid (1.5 g, 88% yield). X-ray quality crystals were grown by slow evaporation from methanol. m.p.: 450 K.

### Refinement

A total of 15 outlier reflections (ΔF^2^/σ(F^2^)>4.5) were removed from the refinment using *OMIT*. There were 57 systematic absence violations involving weak reflections as discussed in the Comment section. The cyclohexanone ring was disordered across two configuraions (see Fig. 1); each was refined with common occupancy factors giving a final ratio a:b of 0.663 (3):0.337 (3). The bond lengths between C16 and C19 to their respective disorder atoms (C17 and C18, a and b) were restrained to be the same using SADI. The bond distances between C20*b* and each of the bound methyl C atoms were similarly restrained.

The methyl H atoms were constrained to an ideal geometry (C—H = 0.98 Å) with *U*_iso_(H) = 1.5*U*_eq_(C), but were allowed to rotate freely about the adjacent C—C bond. All other H atoms were placed in geometrically idealized positions and constrained to ride on their parent atoms with C—H distances of 1.00 (primary), 0.99 (methylene) or 0.95 (phenyl) Å with *U*_iso_(H) = 1.2*U*_eq_(C).

### Crystal data

**Table:**

C_23_H_31_NO	*F*(000) = 736
*M**_r_* = 337.49	*D*_x_ = 1.120 Mg m^−^^3^
Monoclinic, *P*2_1_/*n*	Mo *K*α radiation, λ = 0.71073 Å
Hall symbol: -P 2yn	Cell parameters from 9800 reflections
*a* = 9.7327 (4) Å	θ = 2.4–27.3°
*b* = 17.2187 (6) Å	µ = 0.07 mm^−^^1^
*c* = 12.1303 (4) Å	*T* = 116 K
β = 100.045 (2)°	Block, orange
*V* = 2001.69 (13) Å^3^	0.62 × 0.49 × 0.25 mm
*Z* = 4	

### Data collection

**Table:**

Bruker APEXII CCD diffractometer	4500 independent reflections
Radiation source: fine-focus sealed tube	3690 reflections with *I* > 2σ(*I*)
graphite	*R*_int_ = 0.043
Detector resolution: 8.333 pixels mm^-1^	θ_max_ = 27.3°, θ_min_ = 2.4°
φ and ω scans	*h* = −12→12
Absorption correction: multi-scan (Blessing, 1995)	*k* = −22→22
*T*_min_ = 0.668, *T*_max_ = 0.746	*l* = −15→15
44399 measured reflections	

### Refinement

**Table:**

Refinement on *F*^2^	Primary atom site location: structure-invariant direct methods
Least-squares matrix: full	Secondary atom site location: difference Fourier map
*R*[*F*^2^ > 2σ(*F*^2^)] = 0.050	Hydrogen site location: inferred from neighbouring sites
*wR*(*F*^2^) = 0.134	H atoms treated by a mixture of independent and constrained refinement
*S* = 1.05	*w* = 1/[σ^2^(*F*_o_^2^) + (0.0594*P*)^2^ + 0.720*P*] where *P* = (*F*_o_^2^ + 2*F*_c_^2^)/3
4500 reflections	(Δ/σ)_max_ < 0.001
308 parameters	Δρ_max_ = 0.26 e Å^−^^3^
5 restraints	Δρ_min_ = −0.21 e Å^−^^3^

### Special details

**Table:**

Experimental. ^1^H NMR (500 MHz, CDCl_3_): δ 8.00 (d, 1H, *J* 10 Hz), 7.20-7.17 (m, 2H), 6.93 (t, 1H) 7.71 (d, 1H, *J* 5 Hz), 5.29 (d, 1H, *J* 10Hz), 3.22 (s, 3H), 2.56 (dd, 2H, *J* 5 Hz), 2.32(m, 1H), 2.15 (m, 2H), 1.95 (m, 2H), 1.63 (s, 6H), 0.98 (s, 9H). ^13^C NMR (75 MHz, CDCl_3_): δ 198.5, 165.1, 144.4, 139.5, 134.5, 127.8, 124.1, 121.8, 121.1, 106.9, 91.5, 46.8, 44.9, 39.3, 32.7, 29.7, 28.7, 27.4, 26.8, 24.0. LCMS found: MH^+^ 338.2475; C_23_H_31_NO requires MH^+^ 338.2484; Δ = -2.7 ppm
Geometry. All e.s.d.'s (except the e.s.d. in the dihedral angle between two l.s. planes) are estimated using the full covariance matrix. The cell e.s.d.'s are taken into account individually in the estimation of e.s.d.'s in distances, angles and torsion angles; correlations between e.s.d.'s in cell parameters are only used when they are defined by crystal symmetry. An approximate (isotropic) treatment of cell e.s.d.'s is used for estimating e.s.d.'s involving l.s. planes.
Refinement. Refinement of *F*^2^ against ALL reflections. The weighted *R*-factor *wR* and goodness of fit *S* are based on *F*^2^, conventional *R*-factors *R* are based on *F*, with *F* set to zero for negative *F*^2^. The threshold expression of *F*^2^ > σ(*F*^2^) is used only for calculating *R*-factors(gt) *etc*. and is not relevant to the choice of reflections for refinement. *R*-factors based on *F*^2^ are statistically about twice as large as those based on *F*, and *R*-factors based on ALL data will be even larger.

### Fractional atomic coordinates and isotropic or equivalent isotropic displacement parameters (Å^2^)

**Table:**

	*x*	*y*	*z*	*U*_iso_*/*U*_eq_	Occ. (<1)
O1	0.28457 (12)	0.66246 (6)	0.71213 (10)	0.0437 (3)	
N1	0.79474 (12)	0.49759 (7)	1.02889 (9)	0.0283 (3)	
C1	0.89106 (14)	0.53885 (8)	1.10624 (11)	0.0270 (3)	
C2	1.00469 (15)	0.51059 (9)	1.18053 (12)	0.0331 (3)	
H2	1.0261	0.4567	1.1848	0.040*	
C3	1.08596 (16)	0.56435 (10)	1.24853 (12)	0.0379 (4)	
H3	1.1654	0.5469	1.2995	0.045*	
C4	1.05364 (16)	0.64254 (10)	1.24352 (12)	0.0384 (4)	
H4	1.1103	0.6779	1.2915	0.046*	
C5	0.93804 (15)	0.67029 (9)	1.16829 (12)	0.0320 (3)	
H5	0.9150	0.7239	1.1654	0.038*	
C6	0.85852 (14)	0.61754 (8)	1.09849 (11)	0.0265 (3)	
C7	0.73269 (13)	0.63014 (8)	1.00634 (11)	0.0259 (3)	
C8	0.70101 (13)	0.54643 (8)	0.96512 (11)	0.0262 (3)	
C9	0.79355 (16)	0.41346 (8)	1.02159 (13)	0.0335 (3)	
H9A	0.8428	0.3971	0.9616	0.050*	
H9B	0.6969	0.3950	1.0053	0.050*	
H9C	0.8401	0.3915	1.0929	0.050*	
C10	0.77348 (15)	0.68104 (9)	0.91287 (12)	0.0329 (3)	
H10A	0.8527	0.6574	0.8854	0.049*	
H10B	0.7997	0.7329	0.9425	0.049*	
H10C	0.6940	0.6852	0.8512	0.049*	
C11	0.61232 (15)	0.66848 (9)	1.05326 (12)	0.0340 (3)	
H11A	0.6454	0.7166	1.0921	0.051*	
H11B	0.5790	0.6329	1.1059	0.051*	
H11C	0.5358	0.6803	0.9916	0.051*	
C12	0.60291 (14)	0.52054 (8)	0.87812 (11)	0.0299 (3)	
H12	0.6070	0.4673	0.8587	0.036*	
C13	0.49551 (14)	0.56575 (8)	0.81416 (11)	0.0291 (3)	
H13	0.4886	0.6184	0.8360	0.035*	
C14	0.40147 (14)	0.54095 (8)	0.72454 (11)	0.0286 (3)	
C15	0.29387 (14)	0.59685 (9)	0.67486 (12)	0.0308 (3)	
C16	0.19053 (16)	0.57155 (10)	0.57339 (14)	0.0418 (4)	
H16A	0.0979	0.5924	0.5811	0.063*	
H16B	0.2175	0.5965	0.5068	0.063*	
C19	0.4067 (2)	0.46039 (9)	0.67678 (13)	0.0394 (4)	
H19A	0.496 (2)	0.4472 (12)	0.6680 (17)	0.059*	
H19B	0.375 (2)	0.4235 (12)	0.7229 (18)	0.059*	
C17A	0.1748 (3)	0.48893 (14)	0.5508 (2)	0.0303 (5)	0.663 (3)
H17A	0.118 (3)	0.4799 (16)	0.480 (2)	0.045*	0.663 (3)
H17B	0.129 (3)	0.4671 (16)	0.610 (2)	0.045*	0.663 (3)
C18A	0.3166 (2)	0.45000 (15)	0.5576 (2)	0.0255 (5)	0.663 (3)
H18A	0.366 (3)	0.4800 (15)	0.507 (2)	0.038*	0.663 (3)
C20A	0.3096 (6)	0.3654 (4)	0.5180 (5)	0.0281 (10)	0.663 (3)
C21A	0.4546 (3)	0.32873 (16)	0.5326 (3)	0.0469 (7)	0.663 (3)
H21A	0.4482	0.2777	0.4964	0.070*	0.663 (3)
H21B	0.4919	0.3228	0.6126	0.070*	0.663 (3)
H21C	0.5168	0.3622	0.4982	0.070*	0.663 (3)
C22A	0.2492 (3)	0.36163 (15)	0.39073 (19)	0.0434 (6)	0.663 (3)
H22A	0.1527	0.3805	0.3776	0.065*	0.663 (3)
H22B	0.2511	0.3078	0.3648	0.065*	0.663 (3)
H22C	0.3056	0.3942	0.3496	0.065*	0.663 (3)
C23A	0.2173 (3)	0.31416 (14)	0.5787 (2)	0.0400 (6)	0.663 (3)
H23A	0.2166	0.2610	0.5497	0.060*	0.663 (3)
H23B	0.1219	0.3347	0.5659	0.060*	0.663 (3)
H23C	0.2544	0.3141	0.6591	0.060*	0.663 (3)
C17B	0.2366 (6)	0.4922 (3)	0.5221 (4)	0.0331 (11)	0.337 (3)
H17C	0.1547	0.4715	0.4705	0.050*	0.337 (3)
H17D	0.3085	0.5048	0.4764	0.050*	0.337 (3)
C18B	0.2938 (5)	0.4273 (3)	0.6024 (5)	0.0268 (10)	0.337 (3)
H18B	0.225 (6)	0.421 (3)	0.653 (4)	0.040*	0.337 (3)
C20B	0.3155 (11)	0.3475 (7)	0.5420 (9)	0.030 (2)	0.337 (3)
C21B	0.3865 (6)	0.2909 (3)	0.6310 (5)	0.0512 (15)	0.337 (3)
H21D	0.4037	0.2415	0.5954	0.077*	0.337 (3)
H21E	0.3259	0.2818	0.6863	0.077*	0.337 (3)
H21F	0.4754	0.3128	0.6683	0.077*	0.337 (3)
C22B	0.4077 (6)	0.3596 (3)	0.4542 (5)	0.0498 (15)	0.337 (3)
H22D	0.4241	0.3096	0.4203	0.075*	0.337 (3)
H22E	0.4970	0.3820	0.4897	0.075*	0.337 (3)
H22F	0.3612	0.3950	0.3962	0.075*	0.337 (3)
C23B	0.1757 (5)	0.3156 (3)	0.4890 (5)	0.0526 (16)	0.337 (3)
H23D	0.1150	0.3123	0.5454	0.079*	0.337 (3)
H23E	0.1878	0.2638	0.4589	0.079*	0.337 (3)
H23F	0.1331	0.3501	0.4282	0.079*	0.337 (3)

### Atomic displacement parameters (Å^2^)

**Table:**

	*U*^11^	*U*^22^	*U*^33^	*U*^12^	*U*^13^	*U*^23^
O1	0.0452 (6)	0.0324 (6)	0.0489 (7)	0.0034 (5)	−0.0046 (5)	−0.0071 (5)
N1	0.0312 (6)	0.0245 (6)	0.0280 (6)	−0.0020 (4)	0.0014 (5)	0.0022 (4)
C1	0.0286 (7)	0.0298 (7)	0.0231 (6)	−0.0024 (5)	0.0057 (5)	0.0015 (5)
C2	0.0356 (8)	0.0353 (8)	0.0280 (7)	0.0050 (6)	0.0044 (6)	0.0057 (6)
C3	0.0327 (8)	0.0506 (10)	0.0279 (7)	0.0025 (7)	−0.0018 (6)	0.0026 (6)
C4	0.0362 (8)	0.0451 (9)	0.0311 (7)	−0.0066 (7)	−0.0018 (6)	−0.0062 (6)
C5	0.0327 (7)	0.0315 (7)	0.0313 (7)	−0.0029 (6)	0.0040 (6)	−0.0027 (6)
C6	0.0239 (6)	0.0303 (7)	0.0252 (6)	−0.0007 (5)	0.0040 (5)	0.0022 (5)
C7	0.0250 (6)	0.0247 (7)	0.0271 (6)	−0.0030 (5)	0.0017 (5)	0.0007 (5)
C8	0.0261 (6)	0.0270 (7)	0.0264 (6)	−0.0026 (5)	0.0066 (5)	0.0025 (5)
C9	0.0392 (8)	0.0237 (7)	0.0367 (7)	−0.0017 (6)	0.0046 (6)	0.0022 (6)
C10	0.0331 (7)	0.0302 (7)	0.0342 (7)	−0.0038 (6)	0.0026 (6)	0.0063 (6)
C11	0.0285 (7)	0.0367 (8)	0.0363 (7)	0.0006 (6)	0.0046 (6)	−0.0049 (6)
C12	0.0331 (7)	0.0267 (7)	0.0291 (7)	−0.0051 (5)	0.0033 (6)	−0.0007 (5)
C13	0.0276 (7)	0.0299 (7)	0.0299 (7)	−0.0049 (5)	0.0050 (5)	−0.0018 (5)
C14	0.0297 (7)	0.0292 (7)	0.0263 (6)	−0.0046 (5)	0.0031 (5)	−0.0010 (5)
C15	0.0277 (7)	0.0321 (8)	0.0322 (7)	−0.0048 (5)	0.0040 (6)	−0.0023 (6)
C16	0.0269 (7)	0.0476 (10)	0.0470 (9)	0.0031 (6)	−0.0046 (6)	−0.0127 (7)
C19	0.0522 (10)	0.0295 (8)	0.0313 (8)	0.0017 (7)	−0.0068 (7)	−0.0026 (6)
C17A	0.0213 (12)	0.0320 (12)	0.0342 (13)	0.0011 (10)	−0.0042 (10)	−0.0044 (10)
C18A	0.0219 (11)	0.0286 (13)	0.0249 (12)	−0.0020 (9)	0.0009 (9)	−0.0001 (10)
C20A	0.0269 (14)	0.023 (3)	0.034 (2)	−0.0007 (14)	0.0050 (12)	0.0008 (14)
C21A	0.0356 (13)	0.0441 (15)	0.0596 (18)	0.0067 (11)	0.0046 (12)	−0.0154 (13)
C22A	0.0568 (16)	0.0425 (14)	0.0300 (12)	−0.0024 (11)	0.0047 (11)	−0.0081 (10)
C23A	0.0465 (14)	0.0316 (12)	0.0440 (14)	−0.0074 (10)	0.0137 (11)	−0.0016 (10)
C17B	0.027 (3)	0.036 (3)	0.032 (2)	0.001 (2)	−0.005 (2)	−0.0046 (19)
C18B	0.028 (2)	0.024 (2)	0.029 (2)	−0.0033 (17)	0.0057 (19)	0.0004 (19)
C20B	0.040 (3)	0.013 (5)	0.039 (5)	−0.005 (3)	0.009 (3)	−0.009 (3)
C21B	0.069 (4)	0.028 (3)	0.053 (3)	−0.003 (2)	0.002 (3)	−0.004 (2)
C22B	0.054 (3)	0.049 (3)	0.052 (3)	−0.005 (2)	0.024 (3)	−0.013 (3)
C23B	0.039 (3)	0.049 (3)	0.068 (4)	−0.009 (2)	0.005 (3)	−0.029 (3)

### Geometric parameters (Å, °)

**Table:**

O1—C15	1.2260 (18)	C19—C18B	1.415 (4)
N1—C8	1.3754 (17)	C19—C18A	1.565 (3)
N1—C1	1.3989 (17)	C19—H19A	0.92 (2)
N1—C9	1.4513 (17)	C19—H19B	0.93 (2)
C1—C2	1.3872 (19)	C17A—C18A	1.524 (4)
C1—C6	1.3910 (19)	C17A—H17A	0.95 (3)
C2—C3	1.391 (2)	C17A—H17B	0.98 (3)
C2—H2	0.9500	C18A—C20A	1.531 (7)
C3—C4	1.381 (2)	C18A—H18A	0.99 (3)
C3—H3	0.9500	C20A—C21A	1.528 (6)
C4—C5	1.403 (2)	C20A—C23A	1.537 (5)
C4—H4	0.9500	C20A—C22A	1.554 (7)
C5—C6	1.3828 (19)	C21A—H21A	0.9800
C5—H5	0.9500	C21A—H21B	0.9800
C6—C7	1.5228 (18)	C21A—H21C	0.9800
C7—C11	1.5378 (19)	C22A—H22A	0.9800
C7—C10	1.5389 (19)	C22A—H22B	0.9800
C7—C8	1.5392 (18)	C22A—H22C	0.9800
C8—C12	1.3684 (18)	C23A—H23A	0.9800
C9—H9A	0.9800	C23A—H23B	0.9800
C9—H9B	0.9800	C23A—H23C	0.9800
C9—H9C	0.9800	C17B—C18B	1.523 (7)
C10—H10A	0.9800	C17B—H17C	0.9900
C10—H10B	0.9800	C17B—H17D	0.9900
C10—H10C	0.9800	C18B—C20B	1.587 (14)
C11—H11A	0.9800	C18B—H18B	0.99 (5)
C11—H11B	0.9800	C20B—C23B	1.504 (11)
C11—H11C	0.9800	C20B—C22B	1.521 (11)
C12—C13	1.4204 (19)	C20B—C21B	1.527 (9)
C12—H12	0.9500	C21B—H21D	0.9800
C13—C14	1.3619 (18)	C21B—H21E	0.9800
C13—H13	0.9500	C21B—H21F	0.9800
C14—C15	1.472 (2)	C22B—H22D	0.9800
C14—C19	1.508 (2)	C22B—H22E	0.9800
C15—C16	1.5114 (19)	C22B—H22F	0.9800
C16—C17A	1.452 (3)	C23B—H23D	0.9800
C16—C17B	1.597 (5)	C23B—H23E	0.9800
C16—H16A	0.9900	C23B—H23F	0.9800
C16—H16B	0.9900		
			
C8—N1—C1	111.58 (11)	H16A—C16—H16B	107.1
C8—N1—C9	125.35 (12)	C18B—C19—C14	122.8 (2)
C1—N1—C9	123.04 (11)	C14—C19—C18A	114.15 (14)
C2—C1—C6	122.14 (13)	C18B—C19—H19A	118.0 (13)
C2—C1—N1	128.51 (13)	C14—C19—H19A	111.5 (13)
C6—C1—N1	109.35 (11)	C18A—C19—H19A	104.6 (13)
C1—C2—C3	117.27 (14)	C18B—C19—H19B	78.9 (13)
C1—C2—H2	121.4	C14—C19—H19B	111.0 (13)
C3—C2—H2	121.4	C18A—C19—H19B	106.6 (13)
C4—C3—C2	121.39 (14)	H19A—C19—H19B	108.5 (18)
C4—C3—H3	119.3	C16—C17A—C18A	110.9 (2)
C2—C3—H3	119.3	C16—C17A—H17A	110.8 (17)
C3—C4—C5	120.74 (14)	C18A—C17A—H17A	111.0 (17)
C3—C4—H4	119.6	C16—C17A—H17B	106.3 (16)
C5—C4—H4	119.6	C18A—C17A—H17B	108.2 (17)
C6—C5—C4	118.31 (14)	H17A—C17A—H17B	109 (2)
C6—C5—H5	120.8	C17A—C18A—C20A	114.3 (3)
C4—C5—H5	120.8	C17A—C18A—C19	110.9 (2)
C5—C6—C1	120.13 (12)	C20A—C18A—C19	112.7 (3)
C5—C6—C7	130.43 (13)	C17A—C18A—H18A	105.7 (15)
C1—C6—C7	109.44 (11)	C20A—C18A—H18A	107.5 (15)
C6—C7—C11	110.89 (11)	C19—C18A—H18A	105.0 (15)
C6—C7—C10	110.13 (11)	C21A—C20A—C18A	111.6 (4)
C11—C7—C10	109.83 (12)	C21A—C20A—C23A	108.2 (3)
C6—C7—C8	101.24 (10)	C18A—C20A—C23A	113.2 (5)
C11—C7—C8	113.57 (11)	C21A—C20A—C22A	106.3 (5)
C10—C7—C8	110.91 (11)	C18A—C20A—C22A	109.9 (3)
C12—C8—N1	122.67 (13)	C23A—C20A—C22A	107.5 (4)
C12—C8—C7	128.96 (12)	C18B—C17B—C16	118.4 (4)
N1—C8—C7	108.32 (11)	C18B—C17B—H17A	111.2 (14)
N1—C9—H9A	109.5	C16—C17B—H18A	122.0 (12)
N1—C9—H9B	109.5	C16—C17B—H17C	107.7
H9A—C9—H9B	109.5	H18A—C17B—H17C	122.5
N1—C9—H9C	109.5	C18B—C17B—H17D	107.7
H9A—C9—H9C	109.5	C16—C17B—H17D	107.7
H9B—C9—H9C	109.5	H17A—C17B—H17D	121.5
C7—C10—H10A	109.5	H17C—C17B—H17D	107.1
C7—C10—H10B	109.5	C19—C18B—C17B	105.6 (4)
H10A—C10—H10B	109.5	C19—C18B—C20B	119.4 (5)
C7—C10—H10C	109.5	C17B—C18B—C20B	113.8 (5)
H10A—C10—H10C	109.5	C19—C18B—H18B	100 (3)
H10B—C10—H10C	109.5	C17B—C18B—H18B	106 (3)
C7—C11—H11A	109.5	C20B—C18B—H18B	110 (3)
C7—C11—H11B	109.5	C23B—C20B—C22B	110.5 (8)
H11A—C11—H11B	109.5	C23B—C20B—C21B	109.4 (7)
C7—C11—H11C	109.5	C22B—C20B—C21B	109.4 (8)
H11A—C11—H11C	109.5	C23B—C20B—C18B	109.2 (8)
H11B—C11—H11C	109.5	C22B—C20B—C18B	110.3 (7)
C8—C12—C13	126.21 (13)	C21B—C20B—C18B	107.9 (8)
C8—C12—H12	116.9	C20B—C21B—H21D	109.5
C13—C12—H12	116.9	C20B—C21B—H21E	109.5
C14—C13—C12	126.32 (14)	H21D—C21B—H21E	109.5
C14—C13—H13	116.8	C20B—C21B—H21F	109.5
C12—C13—H13	116.8	H21D—C21B—H21F	109.5
C13—C14—C15	116.93 (13)	H21E—C21B—H21F	109.5
C13—C14—C19	122.16 (13)	C20B—C22B—H22D	109.5
C15—C14—C19	120.91 (12)	C20B—C22B—H22E	109.5
O1—C15—C14	123.00 (13)	H22D—C22B—H22E	109.5
O1—C15—C16	118.98 (13)	C20B—C22B—H22F	109.5
C14—C15—C16	118.03 (13)	H22D—C22B—H22F	109.5
C17A—C16—C15	118.01 (16)	H22E—C22B—H22F	109.5
C15—C16—C17B	111.8 (2)	C20B—C23B—H23D	109.5
C17A—C16—H16A	107.8	C20B—C23B—H23E	109.5
C15—C16—H16A	107.8	H23D—C23B—H23E	109.5
C17B—C16—H16A	132.0	C20B—C23B—H23F	109.5
C17A—C16—H16B	107.8	H23D—C23B—H23F	109.5
C15—C16—H16B	107.8	H23E—C23B—H23F	109.5
C17B—C16—H16B	85.5		
			
C8—N1—C1—C2	−176.44 (14)	C12—C13—C14—C19	−4.0 (2)
C9—N1—C1—C2	5.6 (2)	C13—C14—C15—O1	−1.7 (2)
C8—N1—C1—C6	2.69 (16)	C19—C14—C15—O1	178.83 (15)
C9—N1—C1—C6	−175.30 (13)	C13—C14—C15—C16	178.40 (13)
C6—C1—C2—C3	0.3 (2)	C19—C14—C15—C16	−1.1 (2)
N1—C1—C2—C3	179.35 (13)	O1—C15—C16—C17A	−161.6 (2)
C1—C2—C3—C4	1.0 (2)	C14—C15—C16—C17A	18.3 (3)
C2—C3—C4—C5	−0.7 (2)	O1—C15—C16—C17B	168.3 (3)
C3—C4—C5—C6	−0.8 (2)	C14—C15—C16—C17B	−11.7 (3)
C4—C5—C6—C1	2.0 (2)	C13—C14—C19—C18B	164.7 (3)
C4—C5—C6—C7	−177.19 (14)	C15—C14—C19—C18B	−15.8 (4)
C2—C1—C6—C5	−1.9 (2)	C13—C14—C19—C18A	−164.90 (17)
N1—C1—C6—C5	178.95 (12)	C15—C14—C19—C18A	14.5 (2)
C2—C1—C6—C7	177.53 (12)	C15—C16—C17A—C18A	−48.0 (3)
N1—C1—C6—C7	−1.67 (15)	C16—C17A—C18A—C20A	−171.4 (3)
C5—C6—C7—C11	−59.72 (19)	C16—C17A—C18A—C19	60.0 (3)
C1—C6—C7—C11	120.99 (13)	C14—C19—C18A—C17A	−43.3 (3)
C5—C6—C7—C10	62.06 (19)	C14—C19—C18A—C20A	−172.8 (3)
C1—C6—C7—C10	−117.23 (13)	C17A—C18A—C20A—C21A	−177.1 (3)
C5—C6—C7—C8	179.47 (14)	C19—C18A—C20A—C21A	−49.3 (4)
C1—C6—C7—C8	0.18 (14)	C17A—C18A—C20A—C23A	−54.8 (4)
C1—N1—C8—C12	175.08 (12)	C19—C18A—C20A—C23A	73.0 (4)
C9—N1—C8—C12	−7.0 (2)	C17A—C18A—C20A—C22A	65.3 (4)
C1—N1—C8—C7	−2.53 (15)	C19—C18A—C20A—C22A	−166.9 (3)
C9—N1—C8—C7	175.40 (12)	C15—C16—C17B—C18B	42.5 (6)
C6—C7—C8—C12	−176.03 (14)	C14—C19—C18B—C17B	41.3 (5)
C11—C7—C8—C12	65.07 (18)	C14—C19—C18B—C20B	171.0 (4)
C10—C7—C8—C12	−59.19 (18)	C16—C17B—C18B—C19	−55.4 (6)
C6—C7—C8—N1	1.39 (13)	C16—C17B—C18B—C20B	171.7 (5)
C11—C7—C8—N1	−117.52 (12)	C19—C18B—C20B—C23B	167.3 (5)
C10—C7—C8—N1	118.23 (12)	C17B—C18B—C20B—C23B	−66.8 (7)
N1—C8—C12—C13	174.55 (13)	C19—C18B—C20B—C22B	−71.0 (7)
C7—C8—C12—C13	−8.4 (2)	C17B—C18B—C20B—C22B	54.9 (8)
C8—C12—C13—C14	177.00 (14)	C19—C18B—C20B—C21B	48.5 (8)
C12—C13—C14—C15	176.56 (13)	C17B—C18B—C20B—C21B	174.4 (6)

### Hydrogen-bond geometry (Å, °)

**Table:**

*D*—H···*A*	*D*—H	H···*A*	*D*···*A*	*D*—H···*A*
C5—H5···O1^i^	0.95	2.45	3.3293 (19)	154
C9—H9A···Cg1^ii^	0.98	2.65	3.5705 (17)	156

Symmetry codes: (i) *x*+1/2, −*y*+3/2, *z*+1/2; (ii) −*x*+2, −*y*+1, −*z*+2.
